# Impact of Modified Atmospheres on Growth and Metabolism of Meat-Spoilage Relevant *Photobacterium* spp. as Predicted by Comparative Proteomics

**DOI:** 10.3389/fmicb.2022.866629

**Published:** 2022-06-02

**Authors:** Sandra Fuertes-Perez, Miriam Abele, Christina Ludwig, Rudi F. Vogel, Maik Hilgarth

**Affiliations:** ^1^Lehrstuhl für Technische Mikrobiologie, Technische Universität München, Munich, Germany; ^2^Bayerisches Zentrum für Biomolekulare Massenspektrometrie (BayBioMS), Technische Universität München, Munich, Germany

**Keywords:** *Photobacterium carnosum*, *Photobacterium phosphoreum*, proteomics, modified atmosphere package (MAP), meat spoilage

## Abstract

Modified atmosphere packaging (MAP) is a common strategy to selectively prevent the growth of certain species of meat spoiling bacteria. This study aimed to determine the impact of high oxygen MAP (70% O_2_, 30% CO_2_, red and white meats) and oxygen-free MAP (70% N_2_, 30% CO_2_, also white meat and seafood) on preventing the growth of spoiling photobacteria on meat. Growth of *Photobacterium carnosum* and *P. phosphoreum* was monitored in a meat simulation media under different gas mixtures of nitrogen, oxygen, and carbon dioxide, and samples were taken during exponential growth for a comparative proteomic analysis. Growth under air atmosphere appears optimal, particularly for *P. carnosum*. Enhanced protein accumulation affected energy metabolism, respiration, oxygen consuming reactions, and lipid usage. However, all the other atmospheres show some degree of growth reduction. An increase in oxygen concentration leads to an increase in enzymes counteracting oxidative stress for both species and enhancement of heme utilization and iron-sulfur cluster assembly proteins for *P. phosphoreum*. Absence of oxygen appears to switch the metabolism toward fermentative pathways where either ribose (*P. phosphoreum)* or glycogen (*P. carnosum)* appear to be the preferred substrates. Additionally, it promotes the use of alternative electron donors/acceptors, mainly formate and nitrate/nitrite. Stress response is manifested as an enhanced accumulation of enzymes that is able to produce ammonia (e.g., carbonic anhydrase, hydroxylamine reductase) and regulate osmotic stress. Our results suggest that photobacteria do not sense the environmental levels of carbon dioxide, but rather adapt to their own anaerobic metabolism. The regulation in presence of carbon dioxide is limited and strain-specific under anaerobic conditions. However, when oxygen at air-like concentration (21%) is present together with carbon dioxide (30%), the oxidative stress appears enhanced compared to air conditions (very low carbon dioxide), as explained if both gases have a synergistic effect. This is further supported by the increase in oxygen concentration in the presence of carbon dioxide. The atmosphere is able to fully inhibit *P. carnosum*, heavily reduce *P. phosphoreum* growth *in vitro*, and trigger diversification of energy production with higher energetic cost, highlighting the importance of concomitant bacteria for their growth on raw meat under said atmosphere.

## Introduction

Modified atmosphere packaging (MAP) employs an exchange of the natural atmospheric gas mixture that surrounds a product for a different composition of gases with the aim of prolonging the shelf life a product (McMillin et al., 1999). This method has been used to control the growth of the initial microbiota of raw meat and, consequently, their deteriorating effects for several years (Yam et al., 2005; McMillin, 2008). The meat industry commonly uses oxygen (O_2_), nitrogen (N_2_), and carbon dioxide (CO_2_) on modified atmospheres (Singh et al., 2011) to inhibit bacterial growth on red [O_2_ (70%)/CO_2_ (30%)] and white meat [O_2_ or N_2_ (70%)/CO_2_ (30%)] while maintaining the organoleptic characteristics of raw meat and avoiding consumer rejection (Sante et al., 1994; Eilert, 2005; McKee, 2007; Rossaint et al., 2015). The inhibition or reduction of the growth of diverse spoilage microorganisms benefits the extension of the shelf-life of raw meat and therefore reduces the production of waste derived from the industry.

High O_2_ concentration is used to maintain the bright red color of fresh meat (Taylor et al., 1990; Luño et al., 1998), retard the formation of brown and undesirable metmyoglobin (Mancini and Hunt, 2005), and inhibit strictly anaerobic and microaerobic bacteria (Farber, 1991). It favors formation of superoxide radicals that induce oxidative stress on bacteria (Pan and Imlay, 2001). However, it also promotes the oxidation of lipids on meat and generation of off-odors (Jakobsen and Bertelsen, 2000; Jayasingh et al., 2002). Additional carbon dioxide is used to directly inhibit the growth of aerobic bacteria on fresh meat (Zhao et al., 1994). It is suggested to act by displacing available O_2_, influencing the pH, inducing the structural alteration of the cell membrane, or interfering with the metabolism of the bacteria (Daniels et al., 1985).

Among the bacteria targeted by the use of MAP are photobacteria, typically marine-related symbionts and pathogens of sea animals, also found in seawater suspension and as spoilers of seafood and fish (Dalgaard et al., 1997; Ast and Dunlap, 2005; Urbanczyk et al., 2010; Takahashi et al., 2015; Labella et al., 2017). Some species of the genus *Photobacterium* (*P*.), however, have been found to also colonize and spoil raw meat. Species *P. phosphoreum* and *P. carnosum* have been reported to be relevant microbiota on raw chicken and turkey (Fuertes-Perez et al., 2019), pork (Nieminen et al., 2016), beef (Pennacchia et al., 2011), sausages (Bouju-Albert et al., 2018; Pini et al., 2020), and minced meat (Stoops et al., 2015) (including marinated meat) under multiple gas atmospheres such as air, vacuum, and MAP (high O_2_ and O_2_-absent) (Hilgarth et al., 2018a,b; Fuertes-Perez et al., 2019).

However, the direct effect of MAP on the growth of photobacteria are unclear and the effect of each gas on the relevant species have not yet been studied in detail. Previous research based on metatranscriptomic data of naturally contaminated meat reported little regulation in response to carbon dioxide. The study predicted that the metabolism of photobacteria was not differentially affected by the use of modified atmospheres with or without O_2_ in combination with CO_2_ (Höll et al., 2019). Still, the work aimed at a wider view of the meat microbiota and could not differentiate at the strains/species level. On the other hand, a report based on cell enumeration of photobacteria directly on artificially contaminated meat revealed that a combination of high O_2_ and CO_2_ is, indeed, able to reduce and almost inhibit their growth (Hauschild et al., 2021), but offer no metabolic background to explain the effects of the gas mixture.

Cell enumeration provides an overall idea of the response of bacteria to a specific environmental condition, but the underlying mechanisms of adaptation that these bacteria utilize remain unknown. It is therefore necessary to target the qualitative and quantitative measurement of expressed genes, proteins, and consumed or produced metabolites for said purpose. “Omic” technologies have already been used to unveil the regulation behind the behavior and metabolism of other meat spoiling bacteria (Orihuel et al., 2018; Quintieri et al., 2018; Wang et al., 2018; Kolbeck et al., 2020), leaving still a gap of knowledge for *Photobacterium* spp. and the response of specific strains on meat.

We have monitored the growth of photobacteria *in vitro* under different gas mixtures and followed a comparative proteomics approach in order to determine the direct influence of O_2_ and CO_2_ and their concentration. This study aimed to elucidate the molecular regulations that allow photobacteria to grow and adapt to the packaging conditions using modified atmospheres and also to determine the overall metabolic mechanisms these bacteria use to grow on raw meat. Through the use of proteomics, which is able to depict the enzymatic machinery of the cell, the predictions should allow a closer understanding of their metabolism than previous transcriptomic studies and therefore provide novel insights.

## Materials and Methods

### Bacterial Strains and Pre-Culture

Strains of both species were selected as representative isolates from raw meat. Two strains per species were chosen to cover their previously reported high intra-species variability (Fuertes-Perez et al., 2019, 2021). *P. carnosum* TMW 2.2021^T^ (DSM 105454^T^) is the described type of strain of the species (Hilgarth et al., 2018b) that was isolated from MAP raw chicken meat. Strain TMW 2.2149 was previously isolated from MAP pork (Fuertes-Perez et al., 2019). *P. phosphoreum* strains TMW 2.2103 and TMW 2.2134 were isolated from MAP beef and poultry meat, respectively (Fuertes-Perez et al., 2019).

Strains were inoculated in a pre-culture of meat extract media according to Fuertes-Perez et al. (2019), prepared with 20 g/L meat extract with 20 g/L NaCl and pH 5.8 from the same glycerol stock every time. Pre-cultures were incubated at 15°C overnight in Erlenmeyer flasks for aerobic growth conditions or in gas tight Schott bottles for anaerobic conditions. Cells from the pre-culture were harvested, washed once with 0.85% NaCl (w/v) solution, and re-suspended again in the same solution for further inoculation of the cultures.

### Growth Under Different Gas Atmospheres

Growth of the selected strains was tested on gas tight locked glass bottles filled with 0.4 L of Meat-Simulation-Media (MSM) prepared according to Kolbeck et al. (2019). MSM contains 6% meat extract (w/v) (Merck, Darmstadt, Germany) as the minimum amount at which growth was observed,0.5% glycerol (w/v) (Gerbu Biotechnik GmbH, Heidelberg, Germany), and 0.05 mM Tween80 (Gerbu Biotechnik GmbH, Heidelberg, Germany). Additionally, the media contains 2 μg/ml heminchloride (Roth, Karlsruhe, Germany) dissolved in dimethylsulfoxide (99.8%) (Roth, Karlsruhe, Germany) which was added after autoclaving the media. The pH of the media was adjusted to 5.8 with 100% lactic acid.

Bacteria were inoculated at a start optical density of 0.1 at 600 nm. The growth was monitored by optical density measurement for 48 h or until the stationary phase was reached with constant gas flow, stirring at 120 rpm and at 15°C. Gas mixtures utilized and pumped into the bottles during growth were as follows: (a) air, (b) N_2_ (100%), (c) O_2_/N_2_, (70/30%), (d) N_2_/CO_2_ (70/30%), (e) O_2_/CO_2_/N_2_ (21/30/49%), and (f) O_2_/CO_2_ (70/30%). Samples for proteomic analysis were collected by centrifugation (4,000 xg, 5 min, 4°C) of 100 ml of culture during exponential growth, when the calculated amount of cells was above log 7 CFU ml^−1^. Cells were washed twice with 0.85% NaCl solution, snap-frozen with liquid nitrogen, and stored at −80°C. During the whole sampling process, samples were kept on ice. All experiments were performed in triplicate for each gas atmosphere and each strain.

### Growth Parameters and Statistical Analysis

Optical density measurements obtained for the triplicates of each experiment were used as input for the open source software RStudio (v. 3.3.0) together with the CRAN package grofit (v. 1.1.1-1) to obtain lag-phase (λ), maximum optical density (OD_max_), and maximum growth rate (μ_max_) of the bacteria. Parameters for the analysis were kept as default. Differences between the mean values of each parameter were analyzed with IBM SPSS Statistics v. 28.0 software (IBM Corp., Armonk, NY) by performing one-way analysis of variance (ANOVA) between the gas atmospheres used for each strain, followed by a *post-hoc* Tukey test with a confidence interval set at 95% (*p* < 0.05).

### Preparation of Samples for Proteomic Analysis

Preparation of samples for proteomics analysis was performed with an in-solution sample processing protocol. Shortly, cells were resuspended in urea lysis buffer [8 M urea, 5 mM EDTA, 100 mM ammonium hydrogen carbonate, 1 mM dithiothreitol (DTT), pH 8.0] and mechanically disrupted with acid washed glass beads on a vortex at maximum speed for 10 min at 4°C. The protein concentration was determined by bicinchoninic acid assay (BCA Protein Assay Kit, Thermo Fisher Scientific, US) according to manufacturer's instructions. A total of 20 μg of protein per sample were reduced (10 mM DTT for 30 mins at 30°C) and carbamidomethylated [55 mM chloroacetamide (CAA) for 30 mins at room temperature in darkness]. Digestion of the proteins was carried out by adding trypsin at a 1/100 enzyme/protein ratio (w/w) for 1 h and afterwards by adding another 1/100 enzyme/protein ratio overnight at 37°C.

After digestion, stage tip purification was performed. Therefore, the pH of the samples was measured (pH < 3) with pH strips (MColorpHast, Merck, GER). The in-house built C18 tips using 3 disks (3M) that were equilibrated consecutively with 250 μl 100% acetonitrile (ACN), 250 μl elution solution [40% ACN,0.1% formic acid (FA)], and 250 μl washing solution (2% ACN,0.1% FA) at 1,500 g. Every sample was loaded on the column (5 min at 500 g), and the sample was three times desalted with washing buffer (2% ACN,0.1% FA) for 2 min at 1,500 g. Finally, peptides were eluted with two times 50 μl elution solution (40% ACN,0.1% FA) for 2 min at 500 g. The solvent of all samples was completely subtracted in a centrifugal evaporator (Centrivap Cold Trap −50, Labconco, US) and freshly suspended before MS measurement in washing solution (2% ACN,0.1% FA) before ~0.1 μg of digest was injected into the mass spectrometer per measurement.

### LC-MS/MS Analysis and Data Generation

Liquide chromatography tandem mass spectrometry (LC-MS/MS) measurements were carried out on an Ultimate 3,000 RSLCnano system coupled to a Q-Exactive HF-X mass spectrometer (Thermo Fisher Scientific, US). Full proteome analyses were performed by delivering 0.1 μg of peptides to a trap column (self-packed, ReproSil-pur C18-AQ, 5 mm, Dr. Maisch, 20 mm x 75 mm) at a flow rate of 5 μl/min [High Performance Liquid Chromatography (HPLC) grade water with 0.1% formic acid]. Peptides were transferred to an analytical column (ReproSil Gold C18- AQ, 3 mm, Dr. Maisch, 450 mm 75 mm, self-packed) after 10 min of loading, and separated with a 50 min linear gradient that ranged from 4 to 32% of solvent B [0.1% formic acid in acetonitrile and 5% (v/v) dimethyl sulfoxide (DMSO)] at 300 nl/min flow rate. Both nanoLC solvents contained 5% DMSO to boost MS intensity [solvent A = 0.1% formic acid in HPLC grade water and 5% (v/v) DMSO] (Hahne et al., 2013). The Q-Exactive HF-X mass spectrometer was set in data dependent acquisition (DDA) and positive ionization mode during operation. MS1 spectra (360–1,300 m/z) were recorded at a resolution of 60,000 using a maximum injection time (maxIT) of 45 ms and an automatic gain control (AGC) target value of 3 x 10^6^. In case of the full proteome analyses, up to 18 peptide precursors were selected for fragmentation. Precursors with charge state 2 to 6 were the only ones selected and dynamic exclusion of 25 s was enabled. Higher energy collision-induced dissociation (HCD) and normalized collision energy (NCE) of 26% were used for peptide fragmentation. The precursor isolation window width was set to 1.3 m/z. MS2 Resolution was 15,000 with an AGC target value of 1 x 10^5^ and maximum injection time (maxIT) of 25 ms (full proteome).

### Identification and Quantification of Proteins Using MaxQuant

The software MaxQuant (version 1.6.3.4), with its built-in search engine Andromeda (Cox et al., 2011; Tyanova et al., 2016a), was used to perform peptide identification and quantification. MS2 spectra were searched against the NCBI proteome database of *P. carnosum* TMW 2.2021^T^ (NPIB01), TMW 2.2149 (WMDL01), and *P. phosphoreum* TMW 2.2103 (WMCZ01), TMW 2.2134 (WMCU01), supplemented with common contaminants (built-in option in MaxQuant). Trypsin/P was specified as proteolytic enzyme. Precursor tolerance was set to 4.5 ppm and fragment ion tolerance to 20 ppm. Results were adjusted to 1% false discovery rate (FDR) on peptide spectrum match (PSM) level and protein level by a target-decoy approach that uses reversed protein sequences. A minimal peptide length of seven amino acids was established, and the “match-between-run” function was disabled. Carbamidomethylated cysteine was set as a fixed modification, while oxidation of methionine and N-terminal protein acetylation were set as variable modifications. The proteins, differentially regulated between two growth conditions, were evaluated using the label-free quantification algorithm provided by MaxQuant (LFQ)(Cox et al., 2014). Intensity based absolute quantification (iBAQ) (Schwanhäusser et al., 2011) was carried out to evaluate the expression of proteins within the same sample.

### Statistical Analysis of Proteomic Data and Interpretation of Results

Data processing was performed using the Perseus software (Tyanova et al., 2016b). The workflow included filtering out proteins only identified by site, reverse or from potential contaminants, and performing a log_2_ transformation of the values. We only considered proteins that were detected in at least two out of three replicates in each gas condition. For differential protein analysis, we performed Welch *t*-tests between each pair of gas conditions. Proteins that met the requirements of *p* < 0.05 and log2 fold change > 2 were considered differentially accumulated. Functional annotation of the proteins was obtained from the databases NCBI, Rapid Annotation Subsystem Technology (RAST) server (Aziz et al., 2008), TIGR annotation (Ouyang et al., 2007), and the Kyoto Encyclopedia of Genes and Genomes (KEGG), and manually curated using BLAST.

We performed six different comparisons between conditions to identify the effects of the following: O_2_ (21%) (**I**. air_vs._N_2_); high O_2_ (70%) (**II**. O_2_/N_2__vs._N_2_, **III**. Air_vs._O_2_/N_2_); CO_2_ (30%) under anoxic conditions (**IV**. N_2__vs._N_2_/CO_2_); and CO_2_ under oxic conditions (**V**. N_2_/CO_2__vs._O_2_/CO_2_/N_2_, **VI**. O_2_/CO_2_/N_2__vs._O_2_/CO_2_).

## Results and Discussion

### Overview

#### Growth

**Figure 1:**
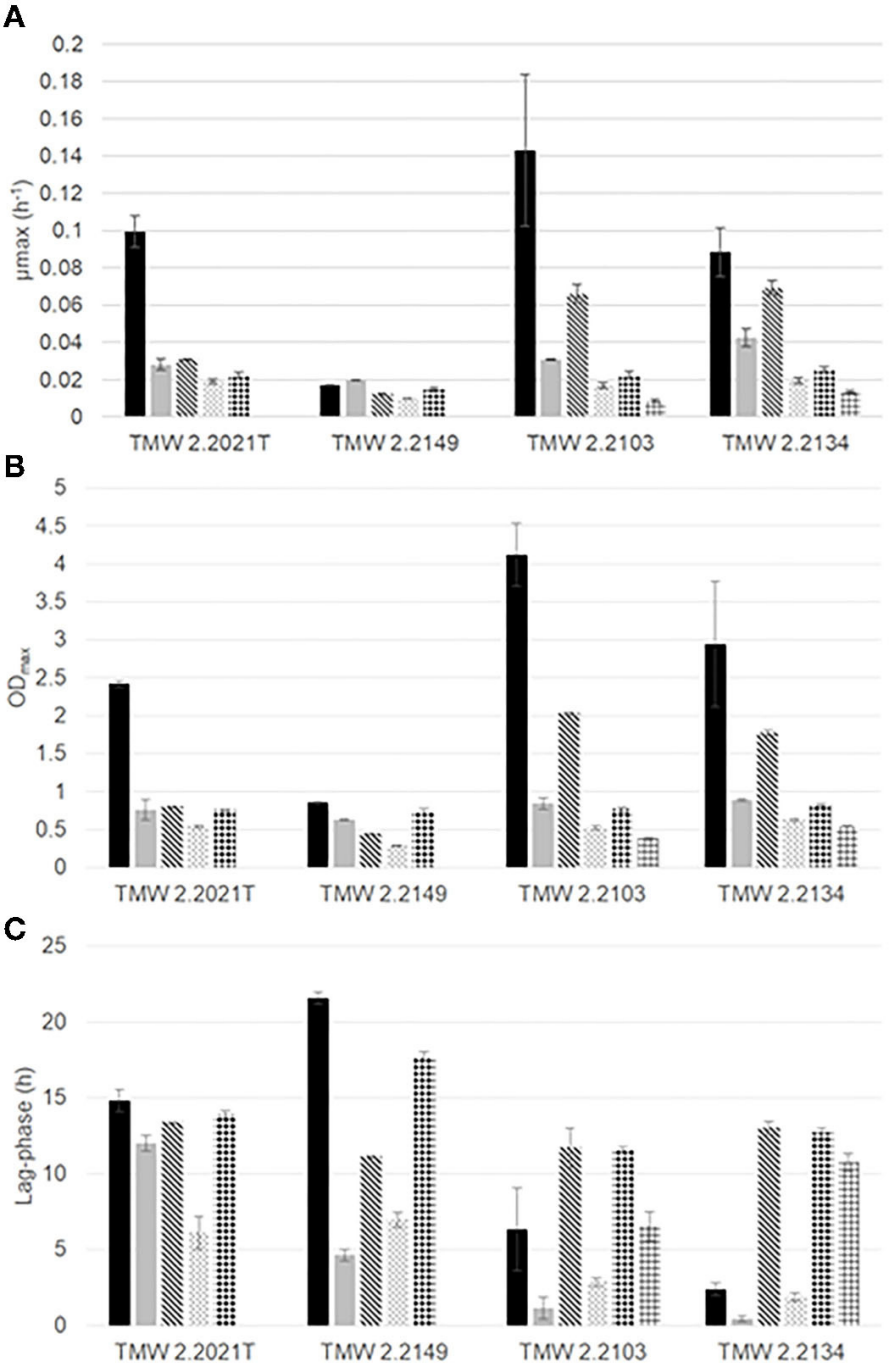
Growth parameters **(A)** maximum growth rate (μ_max_, h^−1^), **(B)** OD_max_, **(C)** lag-phase (h) of *P. carnosum* TMW 2.2021^T^, TMW 2.2149 and *P. phosphoreum* TMW 2.2103, TMW 2.2134 under different gas mixtures: 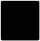 air, 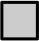 N_2_, 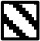 O_2_/N_2_, 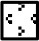 N_2_/CO_2_, 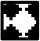 O_2_/CO_2_/N_2_, 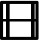 O_2_/CO_2_.

Growth was monitored under different gas mixtures to determine the impact of each gas on the growth parameters of the four strains (μ_max_, OD_max_, lag-phase). All strains were able to grow under five of the six atmospheres tested in this study (air, N_2_, N_2_/CO_2_, O_2_/N_2_, O_2_/CO_2_/N_2_). No growth was observed for *P. carnosum* strains under high O_2_ MAP (O_2_/CO_2_) and therefore only the combined effect of CO_2_ and 21% O_2_ were analyzed. Figure 1 and Supplementary Table 1 contain a representation and summary of growth parameters (μ_max_, OD_max_, lag-phase) for each strain and gas atmosphere. Additionally, Supplementary Figure 1 includes the growth curves for all strains and conditions. Overall *P. phosphoreum* strains have shorter lag-phase, higher maximum growth rates and higher maximum optical density compared to *P. carnosum* consistent with results reported by Fuertes-Perez et al. (2019).

#### Proteomics Analysis

Proteomics analysis was carried out for each strain and growth condition in order to establish a correlation between observed growth dynamics and adaption-driven proteome changes. To visualize the high quality of the proteomics data set and the excellent reproducibility of the different gas atmosphere experiments, we performed an unsupervised hierarchical clustering analysis of all samples (Supplementary Figures 2, 3). All replicates of one gas experiment clustered tightly together. Samples from oxic and anoxic conditions fully separated in the clustering analysis, demonstrating that the highest impact on the cellular proteome of both species arose from the change of aerobic to anaerobic metabolism.

The number of total detected and quantified proteins out of those coded in the genome of each strain was 2,222 (54.7 %) and 2,164 (61.8 %) for *P. carnosum* strains TMW 2.2021^T^ and TMW 2.2149, respectively, and 2,303 (54.3 %) and 2,418 (60.8 %) for *P. phosphoreum* strains TMW 2.2103 and TMW 2.2134, respectively. The effect of each gas on the proteome was determined by comparing differentially accumulated proteins between conditions, as shown in Table 1 for clarification. All proteins differentially accumulated between conditions for each of the strains of photobacteria are displayed in Supplementary Table 2 and the raw data in Supplementary Table 3. We found that between 17 and 119 protein groups for *P. carnosum* and 9 and 126 protein groups for *P. phosphoreum* were differentially accumulated, including up- and downregulated proteins.

**Table 1:** Effects studied and direct comparisons between conditions performed to study them.

**Effect to study**	**Comparison of conditions**	***P. carnosum***		***P. phosphoreum***	
		**TMW 2.2021^T^**	**TMW 2.2149**	**TMW 2.2103**	**TMW 2.2134**
Effect of atmospheric O_2_ concentration (20%/0%)	air_vs_N_2_	78 (27/51)	86 (49/37)	37 (16/21)	56 (28/28)
Effect of high oxygen concentration (70%/0%)	O_2_/N_2__vs_N_2_	119 (59/60)	111 (73/38)	94 (45/49)	91 (40/51)
Effect of high oxygen concentration (21%/70%)	air_vs_O_2_/N_2_	28 (11/17)	43 (19/24)	9 (2/7)	22 (8/14)
Effect of carbon dioxide under anoxic conditions	N_2__vs_N_2_/CO_2_	28 (20/8)	17 (4/13)	48 (32/1)	43 (30/13)
Effect of oxygen concomitant of CO_2_ presence (0%/20% O_2_; high O_2_ MAP)	N_2_/CO_2__vs_O_2_/CO_2_/N_2_	83 (36/47)	68 (47/21)	126 (76/50)	104 (50/54)
Effect of elevated oxygen concomitant of CO_2_ presence (21%/70%; high O_2_ MAP)	O_2_/CO_2_/N_2__vs_O_2_/CO_2_	N.A.	N.A.	126 (21/105)	114 (15/99)

*The total number of proteins differentially accumulated between conditions with the requirements of p-value < 0.05 and log2 fold change >2 are stated for each comparison and each strain. For each value, in green the number of proteins with a higher expression under the first condition, while in red the number of proteins with a higher expression under the second condition. N.A., lack of proteomic data due to complete growth inhibition of the strain*.

### Detection of the Respiratory Chain

**Figure 2:**
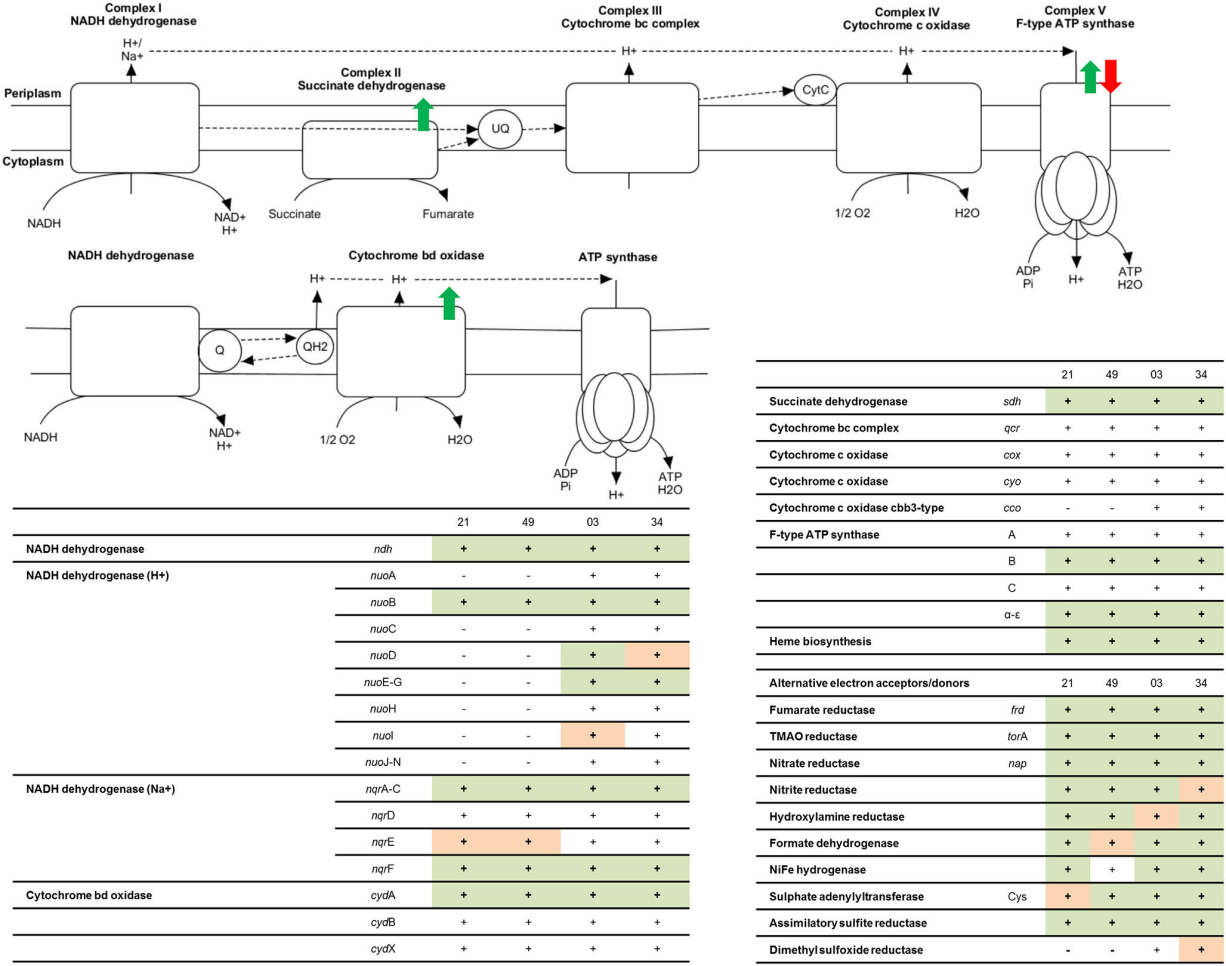
Representation of the functional respiratory chain according to enzymes coded in the genome of photobacteria. Colored arrows represent the regulation points when comparing oxic and anoxic conditions observed: green = higher accumulation under aerobic conditions, compared to anaerobic, red = higher accumulation under anaerobic conditions. The tables include a summary of proteins involved in the respiratory chain for each strain: 21 = *P. carnosum* TMW 2.2021^T^, 49 = *P. carnosum* TMW 2.2149, 03 = *P. phosphoreum* TMW 2.2103, 34 = *P. phosphoreum* TMW 2.2134. − = the gene is not present in the genome of the strain, + (blank) = the gene is present in the genome of the strain but no data for its expression was found in the proteome, + (orange) = the gene is present in the genome of the strain and expression data was found in some of the conditions analyzed, + (green) = the gene is present in the genome of the strain and expression data was found in all conditions analyzed.

According to a comparative genomics study on photobacteria reported by Fuertes-Perez et al. (2021), all strains that were analyzed encode a complete respiratory chain in their genomes. Figure 2 contains an entire summary of genes present in the genomes of each strain and a summary of protein detection. Some of the respiratory enzymes were not detected in the proteomic data of this study. Cytochrome c oxidase (*cox*ABC, *cyo*A-E, *cco*NOP), cytochrome bc complex (*qcr*ABC), and cytochrome b were absent in all strains under all conditions. Specific subunits of come other complexes were also missing, including *nqr*D, *nuo*HJKLMN, *cyd*BX, F0F1 ATP synthase subunit AC, and succinate dehydrogenase subunit CD. While most detected subunits were peripheral, such as ATP synthase subunits α-ε (Jonckheere et al., 2012) and NADH-dehydrogenase sub-units *nuo*EFG (Falk-Krzesinski and Wolfe, 1998), many of the enzymes that were not detected by proteomics in this study were integral membrane proteins (IMPs). IMPs are notoriously challenging proteins for proteomics analyses due to their low solubility when they contain amphipathic structures and their low accumulation levels (Whitelegge, 2013; Jeffery, 2016; Vit and Petrak, 2017).

We detected under all conditions the non-electrogenic NADH dehydrogenase (*ndh*), proton-translocating NADH-dehydrogenase complex subunits *nuo*EFG, Na^+^ translocating NADH-dehydrogenase complex subunits *nqr*ACF, cytochrome bd oxidase subunit *cyd*A, succinate dehydrogenase complex subunits AB, and ATP synthase subunits B and α-ε. The synthesis of an additional proton-translocating NADH dehydrogenase complex by *P. phosphoreum* might influence the efficiency of the respiratory chain and explain, to some extent, the aerobically faster growth of the species in comparison to *P. carnosum*. As predicted before by Fuertes-Perez et al. (2021), they use both the non-electrogenic and sodium-translocating version of Complex I, which is in agreement with the sodium requirement of these bacteria (Hilgarth et al., 2018a,b). Additionally, *P. phosphoreum* also synthesizes the proton-translocating version. We only have evidence of the expression of cytochrome bd oxidase complex. However, it is able to catalyze by itself the complete reduction of O_2_ to water and bypass both complex II and III of the respiratory chain (not detected but present in the genome), coupling the generated proton motive force to the ATP synthesis by the ATP synthase complex (Giuffre et al., 2014), and therefore still functioning as a complete respiratory chain.

### Regulation Toward Presence of Oxygen in Air-Like Condition

**Figure 3:**
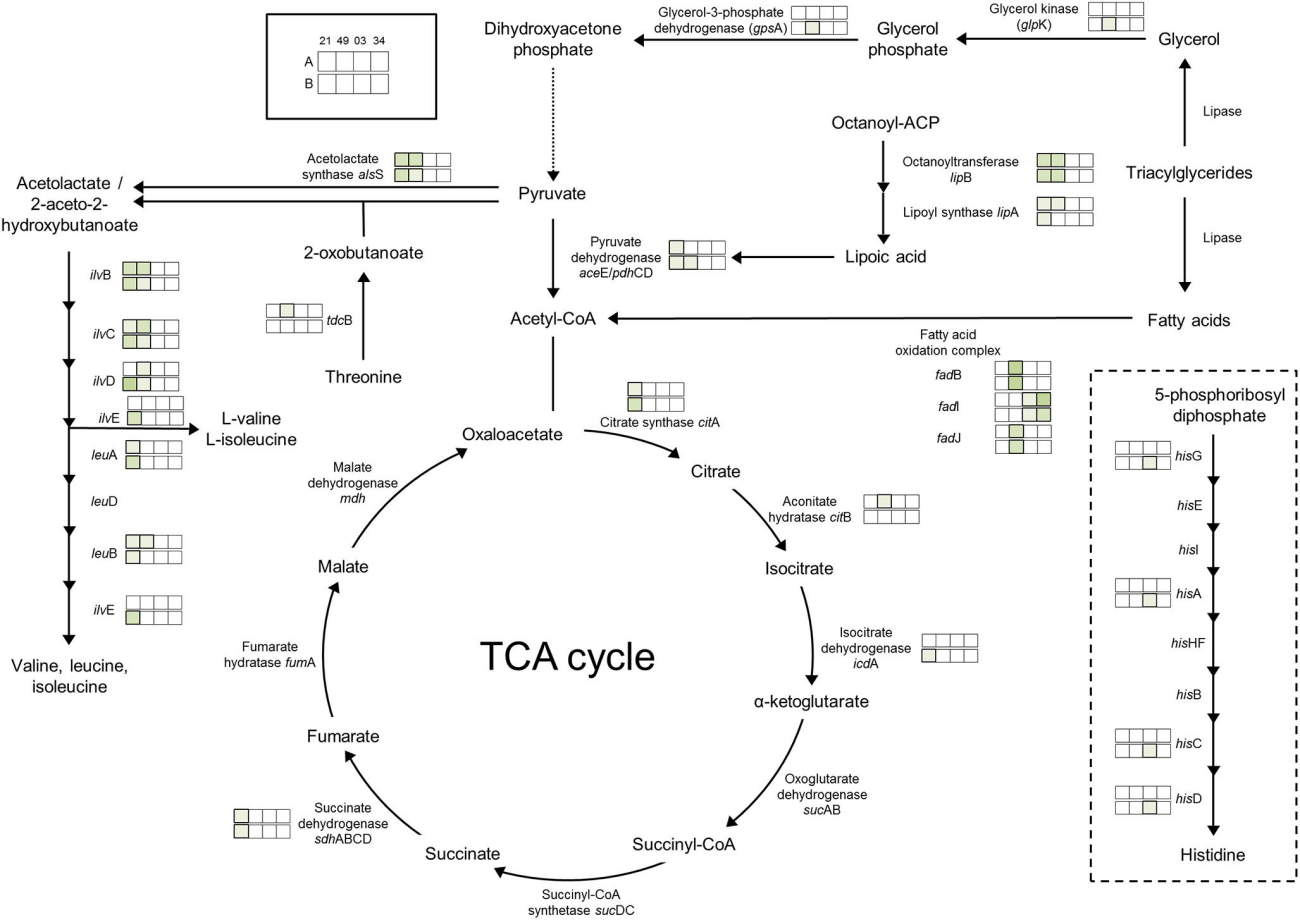
Regulation of enzymes observed under aerobic conditions. The colored boxes display the observed regulation for each strain. Each row of the colored boxes corresponds to one comparison: A. air_vs._N_2_, B. O_2_N_2__vs._N_2_. Each column of the colored boxes corresponds to one strain: *P. carnosum* 21 = TMW 2.2021^T^, 49 = TMW 2.2149, *P. phosphoreum* 03 = TMW 2.2103, 34 = TMW 2.2134. Color code is represented by 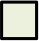 2 log_2_ (diff.), 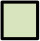 3 log_2_ (diff.), 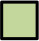 4 log_2_ (diff.), 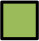 5 log_2_ (diff.), 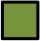 6 log_2_ (diff.), 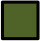 7 log_2_ (diff.), 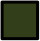 8 log_2_ (diff.).

The effect of the presence of O_2_ (air-like conditions) was determined by growth experiments and the comparison of air_vs._N_2_ conditions (Table 1). The growth of three of the four strains of photobacteria was positively influenced by the presence of O_2_ (21%), with statistically significant (*p* < 0.05) increase of μ_max_ and OD_max_ up to 3 (TMW 2.2021^T^) and 4 times (TMW 2.2103), respectively. Strain TMW 2.2149 showed low growth values in all conditions and displayed improvement of only the OD_max_. Regarding the proteome, *P. carnosum* shows a stronger regulatory response to presence/absence of O_2_ than *P. phosphoreum*, indicating a higher amount of differentially accumulated proteins and adaptive mechanisms to the change in environmental conditions (Figure 3, Table 2).

**Table 2:** Summary of observed pathways/reactions were affected as a consequence of the different gases and concentrations for both species of photobacteria.

	***P. carnosum***	***P. phosphoreum***
Oxygen (21%)	Respiratory chain Oxidoreductase activity Pyruvate metabolism ^*^(TMW 2.2021^T^) TCA cycle Synthesis lipoic acid Oxygen consuming reactions Synthesis of valine, leucine, isoleucine Oxidative stress ^*^(TMW 2.2021^T^) Degradation of fatty acids ^*^(TMW 2.2149)	Oxidoreductase activity Degradation of fatty acids
Oxygen (70%)	Oxidative stress Iron uptake	Oxidative stress Heme utilization/transport Iron-sulfur cluster assembly Histidine biosynthesis ^*^(TMW 2.2103)
Vacuum (N_2_)	Respiratory chain ^*^(TMW 2.2021^T^) Alternative electron acceptors/donors Carbohydrate utilization Peptidases/proteases pH homeostasis Osmoregulation ^*^(TMW 2.2021^T^)	Respiratory chain Alternative electron acceptors/donors Carbohydrate utilization Peptidases/proteases pH homeostasis Osmoregulation
Carbon dioxide	pH homeostasis ^*^(TMW 2.2149) Cellular stress ^*^(TMW 2.2021^T^)	Redox sensing ^*^(TMW 2.2103) Protein S-thiolation ^*^(TMW 2.2134)
Carbon dioxide + Oxygen (21%)	Oxidative stress ^*^(TMW 2.2149) Cellular stress ^*^(TMW 2.2149) pH homeostasis	Oxidative stress Cellular stress pH homeostasis
Carbon dioxide + Oxygen (70%)		Respiratory chain Oxidoreductase activity Alternative electron acceptors/donors Pyruvate metabolism TCA cycle Glyoxylate cycle Fatty acid degradation Amino acids metabolism Heme utilization/transport Iron-sulfur cluster assembly

*Strain-specific regulations are marked with an asterisk and the strain that showed the regulation specified between brackets. Pathway assignation was performed manually*.

Direct adaptation to aerobic conditions are observed in *P. carnosum* by upregulation of respiratory chain enzymes succinate dehydrogenase (TMW 2.2021^T^), cytochrome bd oxidase (TMW 2.2149), and one copy of the ATP-synthase proton pump. Additionally, we detected a slight increase of abundance of enzymes with oxidoreductase activity in the presence of O_2_ for both species to maintain the redox homeostasis of the metabolic machinery. *P. carnosum* accumulated enzymes of the pyruvate oxidation (TMW 2.2021^T^), TCA cycle, and production of lipoic acid under oxic conditions (Figure 3). The lipoic acid is essential as a cofactor for the energy metabolism (including pyruvate dehydrogenase reaction) (Spalding and Prigge, 2010; Solmonson and DeBerardinis, 2018), and serves as an antioxidant against reactive O_2_ species (Packer et al., 1995). The accumulation of proteins affecting mentioned pathways is likely aimed at increasing the energetic yield under aerobic atmosphere in order to enhance growth.

The accumulation of O_2_ consuming acetolactate synthase enzyme in *P. carnosum* that strains under oxic conditions is also interpreted as an adaptive mechanism of the bacteria to the environmental gas atmosphere. In addition, the biosynthesis of valine, leucine, and isoleucine is upregulated under oxic conditions for *P. carnosum* (Figure 3), among the most common amino acids in the proteome of the species (Fuertes-Perez et al., 2021).

Despite detection under all conditions of enzymes superoxide dismutase and catalase/peroxidase in all strains, *P. carnosum* TMW 2.2021^T^ already expressed anti-oxidative stress enzymes with 21% O_2_. Concomitantly, alkyl hydroperoxide reductase, a primary scavenger of hydrogen peroxide in *Escherichia coli* (Seaver and Imlay, 2001), was detected in higher amounts than in anaerobic conditions for the same strain. Unlike *P. phosphoreum*, showing no differential detection, results suggest that *P. carnosum* has a higher sensitivity and, therefore, has an earlier response to stress. This was also supported by previously predicted higher sensitivity to oxidative stress (Fuertes-Perez et al., 2021) and the demonstrated sensitivity to other types of stress such as high pressure, temperature, and salt concentration (Hilgarth et al., 2018b; Hauschild et al., 2020).

Fatty acid oxidation complex subunits had an enhanced accumulation under aerobic conditions: *fad*J, *fad*B for *P. carnosum* TMW 2.2149, and *fad*I for *P. phosphoreum* TMW 2.2103 and TMW 2.2134). This suggests enhanced utilization of lipids under oxic conditions that provides a higher ATP yield (Leverve et al., 2007). As a consequence, photobacteria will contribute to the rancidity during meat spoilage (Mozuraityte et al., 2016) and also provide free fatty acids by lipase activity for other bacteria, leading to accelerated spoilage.

### Regulation Toward Increased Oxygen Concentration

The effects of high O_2_ concentration (70%) could be observed by comparison of growth experiments and the differentially accumulated proteins between the following conditions: I. O_2_/N_2__vs._N_2_ and air_vs._O_2_/N_2_ (Table 1). *P. carnosum* strains show significantly lower μ_max_ and OD_max_ values with a higher O_2_ availability compared to low O_2_ or anoxic conditions. *P. phosphoreum* displays preference for low O_2_ concentrations in all three parameters, but the parameters μ_max_ and OD_max_ show significantly higher values under high O_2_ conditions compared to anaerobic growth of more than 2 times the value. This might be a result of the already suggested higher sensitivity of *P. carnosum* to oxidative stress (Fuertes-Perez et al., 2021). Meanwhile *P. phosphoreum* is able to withstand the stress with minimum growth reduction while benefiting from higher energetic yield of the aerobic metabolism.

Proteins affected by high levels of O_2_ were similar to those observed in air-like conditions and, in many cases, even more enhanced by the increase in O_2_ concentration. The effect of the presence of O_2_ is comparable regardless of concentration of O_2_ on the respiratory chain and pyruvate oxidation for *P. carnosum*, and on the oxidoreductase activity and fatty acid oxidation for both species. Additionally, *P. carnosum* strain TMW 2.2149 showed enhanced glycerol utilization (glycerol kinase *glp*K) with increased O_2_ concentration (Figure 3).

Iron uptake was upregulated for *P. carnosum* strains under high O_2_ conditions for its utilization in heme- and iron-sulfur biosynthesis that is required for aerobic respiration (Paul et al., 2017). On the other hand, heme utilization protein *hut*Z and heme carrier protein *hut*X had a higher accumulation for *P. phosphoreum* strains which are both part of an operon that binds heme and was suggested to act either as storage for said molecule or to facilitate its traffic from the membrane to proteins (Wyckoff et al., 2004). Finally, we found an increase of accumulation in iron-sulfur cluster assembly proteins for *P. phosphoreum* strains, namely, cofactors that are required for several essential pathways such as respiration, carbon metabolism, and protection from oxidizing agents (Mendel et al., 2020).

Additionally, we detected an increase in the response to oxidative stress in both species as an upregulation of several preventive enzymes such as alkyl hydroperoxide reductase, DNA starvation/stationary phase protection protein [linked to protection against multiple types of stress including oxidative (Karas et al., 2015)], thiol peroxidase (prevents membrane lipid oxidation (Cha et al., 2004), and superoxide dismutase, catalase, peroxidase, and thioredoxin [antioxidant activity (Koharyova and Kolarova, 2008)]. *P. phosphoreum* strain TMW 2.2103 also showed upregulation of the histidine biosynthesis pathway (Figure 3), with reported antioxidant and reactive O_2_ species scavenger activities (Wade and Tucker, 1998).

Despite the higher availability of O_2_, growth appears hindered in all cases when comparing optimum growth at air-like conditions and growth under high O_2_ concentration. Results therefore prove that the increase in O_2_ concentration does have an inhibitory effect to some extent in photobacteria, which is most likely derived from the increase in oxidative stress. However, growth was still observed. We conclude that high O_2_ alone is not able to inhibit photobacteria or prevent their growth to spoilage relevant levels.

### Regulation Toward Anaerobic Conditions

Comparisons previously analyzed in order to reveal effect of oxic conditions (air_vs._N_2_ and O_2_/N_2__vs._N_2_) were also the base to determine the effects of growth in absence of O_2_. The lack of O_2_ appears to have an expected detrimental impact on the growth of photobacteria compared to air-like conditions, particularly on the maximum OD_600_ reached, with the aforementioned exception of *P. carnosum* strain TMW 2.2149 and its μ_max_. We found that *P. phosphoreum* does accumulate proteins of the respiratory chain under anoxic conditions, which could suggest a compensatory adaptation of the species to the absence of O_2_ and, therefore, deviation from the higher energetic yield of aerobic respiration. However, it is important to note that the media (as the meat system) used does not contain alternative electron acceptors, such as trimethylamine N-oxide (TMAO), nitrate, or sulfate, predicted to be used by photobacteria (Fuertes-Perez et al., 2021). Therefore, their absence is likely to contribute to the observed growth reduction due to lacking respiratory activity. This idea is supported by Hilgarth et al. (2018b) who reported similar growth of photobacteria under anaerobic and air conditions on marine agar containing nitrate (Hilgarth et al., 2018b). The removal of O_2_ alone is not able to inhibit the growth of photobacteria on meat, but merely limit it (Table 3).

**Table 3:** Summary of the predicted effect of the packaging atmosphere on the growth of photobacteria.

		**Vacuum**			**MAP (no oxygen): white meat**			**MAP (high oxygen, 70%): red meat**		
		**Growth**	**Proteome**	**Expected inhibition**	**Growth**	**Proteome**	**Expected inhibition**	**Growth**	**Proteome**	**Expected inhibition**
*P. carnosum*	TMW 2.2021^T^	+	++	No	+	+	No	N.A.	N.A.	Yes
	TMW 2.2149	+	++	No	+	+	No	N.A.	N.A.	Yes
*P. phosphoreum*	TMW 2.2103	+	+	No	+	+	No	++	++	Yes
	TMW 2.2134	+	+	No	+	+	No	++	++	Yes

*Impact of each gas atmosphere on the growth and proteome of each strain is displayed (- no effect, + moderate effect, ++ strong effect, N.A. no data available). Expected inhibition of growth of photobacteria on meat packages under each atmosphere is included*.

*Growth on air (21% oxygen) is used as the optimum and baseline for the growth comparison with other growth conditions. Effect on the growth is evaluated based on observed reduction as follows: “no effect” indicates no significant difference to optimum conditions, “moderate effect” indicates reduction on the growth parameters but still observed growth, and “strong effect” indicates great reduction of growth parameters. Effect on the proteome was evaluated based on the amount of differentially regulated proteins*.

There is an enhanced accumulation of one gene copy of the ATP-synthase proton pump for *P. phosphoreum* strains (also observed for *P. carnosum* TMW 2.2021^T^), while the other copy was accumulated only for *P. carnosum* under oxic conditions. Gene duplication in this case might respond to an environmental adaptive strategy, with one copy serving as the main proton pump in optimal oxic conditions, and the other as a compensatory copy under anoxic atmospheres (Kondrashov, 2012).

**Figure 4:**
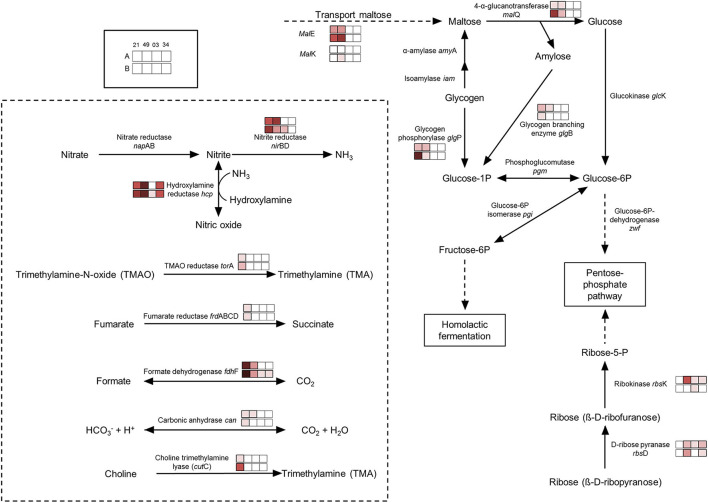
Regulation of enzymes observed under anaerobic conditions. The colored boxes display the observed regulation for each strain. Each row of the colored boxes corresponds to one comparison: A. air_vs._N_2_, B. O_2_N_2__vs._N_2_. Each column of the colored boxes corresponds to one strain: *P. carnosum* 21 = TMW 2.2021^T^, 49 = TMW 2.2149, *P. phosphoreum* 03 = TMW 2.2103, 34 = TMW 2.2134. Color code is represented by 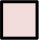 −2 log_2_ (diff.), 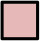 −3 log_2_ (diff.), 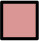 −4 log_2_ (diff.), 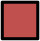 −5 log_2_ (diff.), 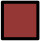 −6 log_2_ (diff.), 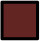 −7 log_2_ (diff.), 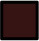 −8 log_2_ (diff.).

There is an accumulation of enzymes involved in the use of alternative electron acceptors/donors in both species, which is particularly stronger in *P. carnosum* strains, many of which were detected under all conditions. We detected upregulation in some of the strains of trimethylamine-N oxide reductase, fumarate reductase, and nitrite reductase in addition to formate dehydrogenase (Figure 4). In particular, nitrite reductase, formate dehydrogenase, and hydroxylamine reductase had a higher accumulation than the rest on the four strains analyzed. Formate is used by bacteria as alternative electron donor and is coupled to the reduction of electron acceptors such as fumarate or nitrate (Ferry, 1990). In addition, cytochrome c (*nap*C/*nir*T) family protein was also only detected under anoxic conditions for strain TMW 2.2021^T^ and was previously reported as mediator during anaerobic respiration with nitrate or nitrite using formate as electron donor (Simon et al., 2000). Results suggest that photobacteria use more than one type of electron acceptor during anaerobic respiration. Results also suggest that nitrate/nitrite and formate might be the preferred redox couple. Nitrite and nitrate are both compounds commonly used in meat preservation, even in the European Union, mostly commonly on cured meats. In raw unprocessed meat, the natural availability of nitrate/nitrite is very low, impairing the anaerobic respiration by means of said compounds (Ferysiuk and Wójciak, 2020). However, both nitrate and nitrite are common in water, and their use might be a remaining conserved feature from the common lifestyle of photobacteria as marine bacteria.

Both species show an accumulation of enzymes involved in the use of carbohydrates under anoxic conditions resulting from the lack of O_2_ and alternative electron acceptors (no respiration) and the switch to fermentative/sugars utilization pathways. Still, both species also appear to have different preferences for the carbohydrate itself. *P. carnosum* strains heavily increase the expression of glycogen and maltose degradation/transport pathways (Figure 4). *P. phosphoreum* strains, on the other hand, due to lack of glycogen and maltose utilization enzymes, regulated mainly the ribose metabolism. Glycogen, ribose, and maltose can be commonly found on raw meat with average values of 1.87 g/kg, 0.5-1 mmol/kg, and 0.02-0.2 mmol/kg, respectively (Koutsidis et al., 2008a,b). The ability of *P. carnosum* to metabolize the three sugars, in contrast to *P. phosphoreum* which is only able to utilize ribose, had already been reported (Fuertes-Perez et al., 2021) together with the production of acid from their utilization (Fuertes-Perez et al., 2019).

In addition to the utilization of carbohydrates, we also observed an accumulation of unspecific peptidases/proteases on both species. The lack of alternative electron acceptors present in the media hinders anaerobic respiration, reducing the energetic yield and, in turn, might enhance the diversification of carbon sources in order to increase the total energy output.

We observed an enhanced accumulation of a battery of enzymes involved in pH balance and alkalization that might represent a response to acidification of the media during carbohydrate fermentation, as previously reported by Fuertes-Perez et al. (2019). The carbonic anhydrase (only *P. carnosum* strains) helps maintain pH homeostasis by interconverting CO_2_ and acid (Occhipinti and Boron, 2019). Both the nitrite reductase and hydroxylamine reductase from nitrogen metabolism are accumulated on both species and are able to produce ammonia from nitrite or hydroxylamine to increase the pH (Figure 4). To a lesser extent, we observed an increase in accumulation of choline trimethylamine-lyase (*cut*C) in one strain (TMW 2.2021^T^) of *P. carnosum* which is able to deaminate choline into trimethylamine (TMA). Choline can be found on raw meat at average levels of 0.7 mg/g (Lewis et al., 2015), while TMA is one of the main spoilage products generated by photobacteria on fish (Dalgaard, 1995). We also observed enhanced detection of anaerobic glycerol-3-phosphate dehydrogenase on *P. phosphoreum* strains and *P. carnosum* strain TMW 2.2021^T^ that catalyzes the production and accumulation of glycerol and helps maintain osmoregulation during osmotic stress conditions (Albertyn et al., 1994).

### Impact of Carbon Dioxide Under Anaerobic Conditions

The comparison of anoxic conditions with and without addition of CO_2_ (N_2__vs._N_2_/CO_2_) allows the determination of the direct effect of CO_2_ alone on analyzed photobacteria. In terms of growth, it appears to negatively impact the growth of photobacteria by significantly decreasing the μ_max_ and the OD_max_ of all strains in comparison to the rest of conditions (except O_2_/CO_2_).

Most pathways appear unaffected under anoxic conditions when comparing the presence and absence of CO_2_. No common strategy to the strains of each species that was specific to high environmental levels of CO_2_ rather than a response to anaerobic metabolism was identified to counteract the presence of CO_2_. We only observed strain-specific regulations of single enzymes, such as an increase of trimethylamine-N-oxide reductase (*tor*A) enzyme in *P. carnosum* TMW 2.2149, producing trimethylamine and contributing to alkalization. *P. carnosum* TMW 2.2021T strain showed higher regulation of cellular stress proteins. In addition, *P. phosphoreum* strains TMW 2.2103 and TMW 2.2134 also showed higher accumulation of the glutathione-S-transferase and bifunctional glutathionylspermidine amidase/synthase enzymes (involved in redox sensing and protein S-thiolation) (Pai et al., 2011), respectively, in presence of carbon dioxide.

In conclusion, photobacteria do not show a common adaptation to environmental presence of carbon dioxide alone. We suggest that photobacteria do adapt to CO_2_/acidification as a response to their own metabolism and presence/absence of O_2_, rather than sensing the environmental levels of CO_2_. Consequently, the higher concentration of CO_2_ might increase the adverse effect on the bacteria and, since no adaptation to increased stress is performed to counteract the detrimental effect of CO_2_, the growth is negatively affected. Still, the modified atmosphere (N_2_/CO_2_ 70/30%) is unable to prevent the growth of photobacteria (Table 3).

### Proposed Synergistic Effect of Oxygen and Carbon Dioxide

The effects of combined CO_2_ and O_2_ at air-like conditions were determined by the comparison N_2_/CO_2__vs._O_2_/CO_2_/N_2_ and considering the effects of aerobic vs. anaerobic conditions without the presence of CO_2_. Presence of air-like O_2_ concentration when CO_2_ is present appears to benefit the growth (μ_max_ and OD_max_) of all strains when compared to the sole presence of CO_2_ anaerobically. However, it is only statistically significant for *P. carnosum* strains, namely, μ_max_ of TMW 2.2149 and OD_max_ of both strains.

When O_2_ is once again introduced to the gas mixture in the presence of carbon dioxide, similar regulations are observed as when CO_2_ was absent (air vs. N_2_). There is an enhancement of oxidoreductase activity, transport of iron, and other metals that might be required for the synthesis of cofactors, pyruvate oxidation, synthesis of lipoic acid, TCA cycle and fatty acid degradation for *P. carnosum* strains, and enhancement of heme utilization proteins, oxidoreductase activity, iron transport, and assembly of iron-sulfur clusters for *P. phosphoreum*. Additionally, we observed an increase in oxidative stress and cellular stress proteins on *P. phosphoreum* strains and *P. carnosum* TMW 2.2149 strain. The induction of oxidative stress response was absent from most strains when the O_2_ concentration was still 21%, but it appears enhanced when the comparison is made in presence of CO_2_. These results might suggest a synergistic effect between CO_2_ and O_2_ that emulates the effects of high O_2_ concentrations even at low O_2_ percentages (21%) when CO_2_ is present. The enhanced effect of the lower O_2_ concentration might be tied to the suggested disruptive mechanism of action of CO_2_ over the cell membrane (Daniels et al., 1985), allowing a faster diffusion of O_2_ into the cell, thereby emulating the effects of the higher O_2_ concentration.

We also observed a reduction of accumulation of acid-counteracting reactions as a response to aerobic growth even in presence of CO_2_, supporting the idea that photobacteria do not sense the environmental levels of carbon dioxide. The enzyme hydroxylamine reductase had a lower accumulation in presence of O_2_ for *P. carnosum* strains, and so did the enzyme carbonic anhydrase for *P. carnosum* strain TMW 2.2021^T^. The lysine decarboxylase, with lower accumulation levels for *P. phosphoreum* strains in the presence of O_2_ catalyzes the proton-dependent decarboxylation of L-lysine to produce the polyamine cadaverine. It also plays a role in pH homeostasis by consuming protons and neutralizing the acidic by-products of carbohydrate fermentation (Moreau, 2007). The enzyme glutamate decarboxylase, also with a lower accumulation for *P. phosphoreum* strains, is reported as one of the most efficient methods for growth under acidic conditions via production of γ-aminobutyrate (GABA) for *Lysteria monocytogenes* (Cotter et al., 2005).

On a note on the spoilage potential of the species, amino acid decarboxylases encoded in the genome of each strain were detected under all conditions. Enzymes arginine decarboxylase (L-arginine to agmatine and CO_2_), agmatinase (agmatine to putrescine and urea), and glutamate decarboxylase (L-glutamate to GABA and CO_2_) were detected on all conditions for all four strains. Additionally, both *P. phosphoreum* strains accumulated under all conditions the tyrosine decarboxylase (L-tyrosine to tyramine and CO_2_) and lysine decarboxylase (L-lysine to cadaverine and CO_2_). Results revealed that regardless of the atmosphere used, photobacteria are able to produce a wide range of biogenic amines and contaminate the raw meat upon growth, as previously predicted by transcriptomics analysis before (Höll et al., 2019).

### Impact of High Oxygen and Carbon Dioxide

The effects of the increase in O_2_ concentration (up to 70%) compared to air-like conditions in the presence of CO_2_ were studied by the comparison O_2_/CO_2_/N_2__vs._O_2_/CO_2_ and by considering the effects of increased O_2_ concentration alone. The increase in O_2_ concentration when CO_2_ is present significantly impacts the growth of photobacteria by fully inhibiting *P. carnosum* and decreasing the growth rate and maximum OD of both *P. phosphoreum* strains (Figure 1 and Table 3).

Response to oxidative stress between high and low O_2_ in presence of CO_2_ was the same, contrary to observed results between the same conditions in absence of CO_2_. These results again support the idea of a synergistic effect of CO_2_ and O_2_, and that the response of photobacteria to oxidative stress already reaches its peak with low O_2_ concentrations rather than with 70%.

The increase of O_2_ concentration in the gas mixture induces the accumulation of multiple proteins on both strains of *P. phosphoreum* when compared to the low O_2_ mixture. The response of both strains appears to be the enhancement of most pathways and reactions in the cells, including the respiratory chain, oxidoreductase activity, alternative electron acceptors and donors, pyruvate metabolism, TCA cycle, glyoxylate cycle, fatty acid degradation, and amino acid metabolism. The response observed in both strains suggests that the combination of high O_2_ and CO_2_ in the gas mixture is enough to override the stress response of the bacteria. While it is not possible to determine the specific response of *P. carnosum* due to its lack of growth, we suggest that *P. phosphoreum* enters a state where survival is prioritized. The species upregulates the entire metabolic machinery as a “panic” reaction against extreme environmental stress. The species might trade off the energy required to maintain such a large enzymatic range for the diversification of energy production or several adaptive mechanisms. The observed growth parameters also suggest that this trade-off might allow the photobacteria to survive under high stress conditions such as those derived from the combined action of high O_2_ concentration (oxidative), carbon dioxide (osmotic) and their synergia. However, in said cases, growth is severely hindered due to an energetic yield being either very low or null.

We observed, however, that proteins related to the heme utilization and iron-sulfur cluster assembly were significantly less accumulated in conditions with high O_2_ and CO_2_ compared to low O_2_ and CO_2_. This is contrary to what is observed between the two conditions in the absence of CO_2_. This phenomenon might be an indication that photobacteria are not able to efficiently use O_2_ in this gas mixture despite its higher percentage, and therefore do not fully benefit from the higher yield of aerobic metabolism.

Despite previous reports supporting the reduced growth of photobacteria under modified atmospheres in packaged raw meat (Hauschild et al., 2021), it is still a common niche from which these species are isolated (Fuertes-Perez et al., 2019) in high cell numbers of >10^8^ log CFU/g. While we deliberately chose to study specific strains alone *in vitro* in this study without any interference or bias by a consortium or variations of substrates, differences in observed growth might be due to the presence of other species of meat spoilers or differences in the model used for growth compared to naturally contaminated raw meat. Spoilage species can have an influence by consumption of O_2_ and by reduction of part of the stress induced, as in is the case of *B. thermosphacta* (Kolbeck et al., 2019), or commensal relationships with photobacteria (Hauschild et al., 2021; Hauschild, 2022). Additionally, the model used in this study, due to the limitations in proteomic sample collection, requires planktonic growth with constant shaking reducing the formation of protective strategies, such as biofilms, that modify diffusion of gases to the cells (Flemming, 1993).

## Conclusion

We have demonstrated that both species of photobacteria commonly found on raw meat appear to be influenced by the gas mixture that surrounds them, both in their growth and in proteome regulation. Changes in the surrounding gas mixture are, in most cases, unable to inhibit them. Therefore, both species are still able to grow and adapt to variations of the atmosphere composition. Photobacteria are able to withstand the lack of O_2_, increase of O_2_ concentration, and presence of CO_2_ alone, and are therefore able to colonize raw meat under those circumstances and spoil it. On the other hand, modified atmospheres containing both high concentrations of O_2_ and CO_2_ prove to be effective in preventing and limiting their growth. However, the reported presence of both species on raw meat packed under said gas mixture suggests that photobacteria might not be lone-wolfs and that they are likely dependent on the presence of concomitant bacteria to lessen the stress caused by both gases.

## Data Availability Statement

The data presented in the study are deposited in the PRIDE repository (https://www.ebi.ac.uk/pride/archive/), accession number PXD031343.

## Author Contributions

SF-P: conceptualization, data curation, formal analysis, investigation, methodology, validation, visualization, and writing-original draft. MA: mass spectrometric analysis, quality control, validation, and writing-editing and review. CL: proteomic conceptualization, quality control, supervision, and writing-editing and review. MH: project administration, funding acquisition, conceptualization, supervision, and writing-editing and review. RV: project administration, funding acquisition, conceptualization, supervision, resources, and writing-editing and review. All authors contributed to the article and approved the submitted version.

## Funding

Part of this work was funded by the Federal Ministry for Economic Affairs and Climate Action (BMWK) *via* the German Federation of Industrial Research Associations (AiF) and the Industry Association for Food Technology and Packaging (IVLV), project number AiF 20113N1. CL and MA were supported by EPIC-XS, project number 823839 and funded by the Horizon 2020 program of the European Union.

## Conflict of Interest

The authors declare that the research was conducted in the absence of any commercial or financial relationships that could be construed as a potential conflict of interest.

## Publisher's Note

All claims expressed in this article are solely those of the authors and do not necessarily represent those of their affiliated organizations, or those of the publisher, the editors and the reviewers. Any product that may be evaluated in this article, or claim that may be made by its manufacturer, is not guaranteed or endorsed by the publisher.
