# Supplementary material for: Impact of Modified Atmospheres on Growth and Metabolism of Meat-Spoilage Relevant Photobacterium spp. as Predicted by Comparative Proteomics
Source: Front Microbiol. 2022 Jun 2;13:866629. doi: 10.3389/fmicb.2022.866629 (PMC9201721; doi:10.3389/fmicb.2022.866629)
Supplement: Supplementary file 1 [file Table_1.DOCX]

**Table S1.** Growth parameters of all strains under different gas atmospheres on meat simulation media.

|  | Lag-phase (h) | SE (h) | OD_max_ | SE | μ_max_ (division/h) | SE (division/h) |
| --- | --- | --- | --- | --- | --- | --- |
| *P. carnosum* TMW 2.2021^T^ |  |  |  |  |  |  |
| Air | 14.83^b,c,d^ | 0.74 | 2.42^b,c,d,e^ | 0.05 | 0.0994^b,c,d,e^ | 0.0084 |
| N_2_ | 12.02^a,d,e^ | 0.51 | 0.76^a,d^ | 0.14 | 0.0281^a,d^ | 0.0029 |
| O_2_/N_2_ | 13.36^a,d,e^ | 0.03 | 0.8^a,d^ | 0.01 | 0.0308^a,d,e^ | 0.0003 |
| N_2_/CO_2_ | 6.08^a,b,c,e^ | 1.09 | 0.54^a,b,c,e^ | 0.02 | 0.0191^a,b,c^ | 0.0012 |
| O_2_/CO_2_/N_2_ | 13.9^b,c,d^ | 0.25 | 0.75^a,d^ | 0.01 | 0.0225^a,c^ | 0.0016 |
| O_2_/CO_2_ | N.A. | N.A. | N.A. | N.A. | N.A. | N.A. |
| *P. carnosum* TMW 2.2149 |  |  |  |  |  |  |
| Air | 21.57^b,c,d,e^ | 0.38 | 0.85^b,c,d,e^ | 0.02 | 0.0171^b,c,d,e^ | 0.0002 |
| N_2_ | 4.64^a,c,d,e^ | 0.39 | 0.63^a,c,d,e^ | 0.01 | 0.0198^a,c,d,e^ | 0.0002 |
| O_2_/N_2_ | 11.12^a,b,d,e^ | 0.05 | 0.44^a,b,d,e^ | 0.01 | 0.0121^a,b,d,e^ | 0.0004 |
| N_2_/CO_2_ | 6.96^a,b,c,e^ | 0.50 | 0.29^a,b,c,e^ | 0.01 | 0.0098^a,b,c,e^ | 0.0001 |
| O_2_/CO_2_/N_2_ | 17.73^a,b,c,d^ | 0.33 | 0.74^a,b,c,d^ | 0.04 | 0.0147^a,b,c,d^ | 0.0013 |
| O_2_/CO_2_ | N.A. | N.A. | N.A. | N.A. | N.A. | N.A. |
| *P. phosphoreum* TMW 2.2103 |  |  |  |  |  |  |
| Air | 6.35^b,c,d,e^ | 2.73 | 4.12^b,c,d,e^ | 0.42 | 0.143^b,c,d,e,f^ | 0.0408 |
| N_2_ | 1.17^a,c,e,f^ | 0.73 | 0.84^a,c,d,f^ | 0.07 | 0.0309^a,c,f^ | 0.0005 |
| O_2_/N_2_ | 11.71^a,b,d,f^ | 1.30 | 2.03^a,b,d,e,f^ | 0.02 | 0.0662^a,b,d,e,f^ | 0.0048 |
| N_2_/CO_2_ | 2.87^a,c,e,f^ | 0.27 | 0.52^a,b,c,f^ | 0.03 | 0.0169^a,c,f^ | 0.0014 |
| O_2_/CO_2_/N_2_ | 11.62^a,b,d,f^ | 0.17 | 0.76^a,c,f^ | 0.03 | 0.0226^a,c,f^ | 0.0018 |
| O_2_/CO_2_ | 6.54^b,c,d,e^ | 0.98 | 0.38^b,c,d,e^ | 0.02 | 0.0086^a,b,c,d,e^ | 0.0009 |
| *P. phosphoreum* TMW 2.2134 |  |  |  |  |  |  |
| Air | 2.41^b,c,d,e,f^ | 0.39 | 2.94^b,c,d,e,f^ | 0.83 | 0.0884^b,c,d,e,f^ | 0.0130 |
| N_2_ | 0.45^a,c,d,e,f^ | 0.21 | 0.89^a,c,f^ | 0.01 | 0.0424^a,f^ | 0.0048 |
| O_2_/N_2_ | 13.02^a,b,d,f^ | 0.44 | 1.77^a,b,d,e,f^ | 0.04 | 0.0694^a,f^ | 0.0037 |
| N_2_/CO_2_ | 1.89^a,b,c,e,f^ | 0.25 | 0.63^a,c,f^ | 0.02 | 0.0194^a,f^ | 0.0016 |
| O_2_/CO_2_/N_2_ | 12.67^a,b,d,f^ | 0.35 | 0.83^a,c,f^ | 0.02 | 0.0253^a,f^ | 0.0015 |
| O_2_/CO_2_ | 10.74^a,b,c,d,e^ | 0.60 | 0.54^a,b,c,d,e^ | 0.00 | 0.0134^a,b,c,d,e^ | 0.0008 |

Displayed numbers represent average values and standard errors obtained from the three independent replicates. The superscript letters indicate significant differences between conditions according to a confidence interval of 95% (p-value 0.05): **a**. Air; **b**. N_2_; **c.** O_2_/N_2_; **d.** N_2_/CO_2_; **e.** O_2_/CO_2_/N_2_; **f.** O_2_/CO_2_.

**Table S2.** Complete list of proteins differentially accumulated between conditions for the four strains. Pc = *P. carnosum*, Pp = *P. phosphoreum*. Proteins that were detected as differentially accumulated between conditions for the strains are listed together with ORF identification, annotation, and the calculated protein fold-changes. The table itself is presented in a separate Excel file, with one tab for each strain.

**Table S3.** Raw data from LC-MS/MS measurements that served to calculate the protein-fold changes for each strain and condition. The table itself is presented in a separate Excel file, with one tab for each strain.
