# Supplementary material for: Impact of Modified Atmospheres on Growth and Metabolism of Meat-Spoilage Relevant Photobacterium spp. as Predicted by Comparative Proteomics
Source: Front Microbiol. 2022 Jun 2;13:866629. doi: 10.3389/fmicb.2022.866629 (PMC9201721; doi:10.3389/fmicb.2022.866629)
Supplement: Supplementary file 4 [file Presentation_1.PPTX]

## Slide 1
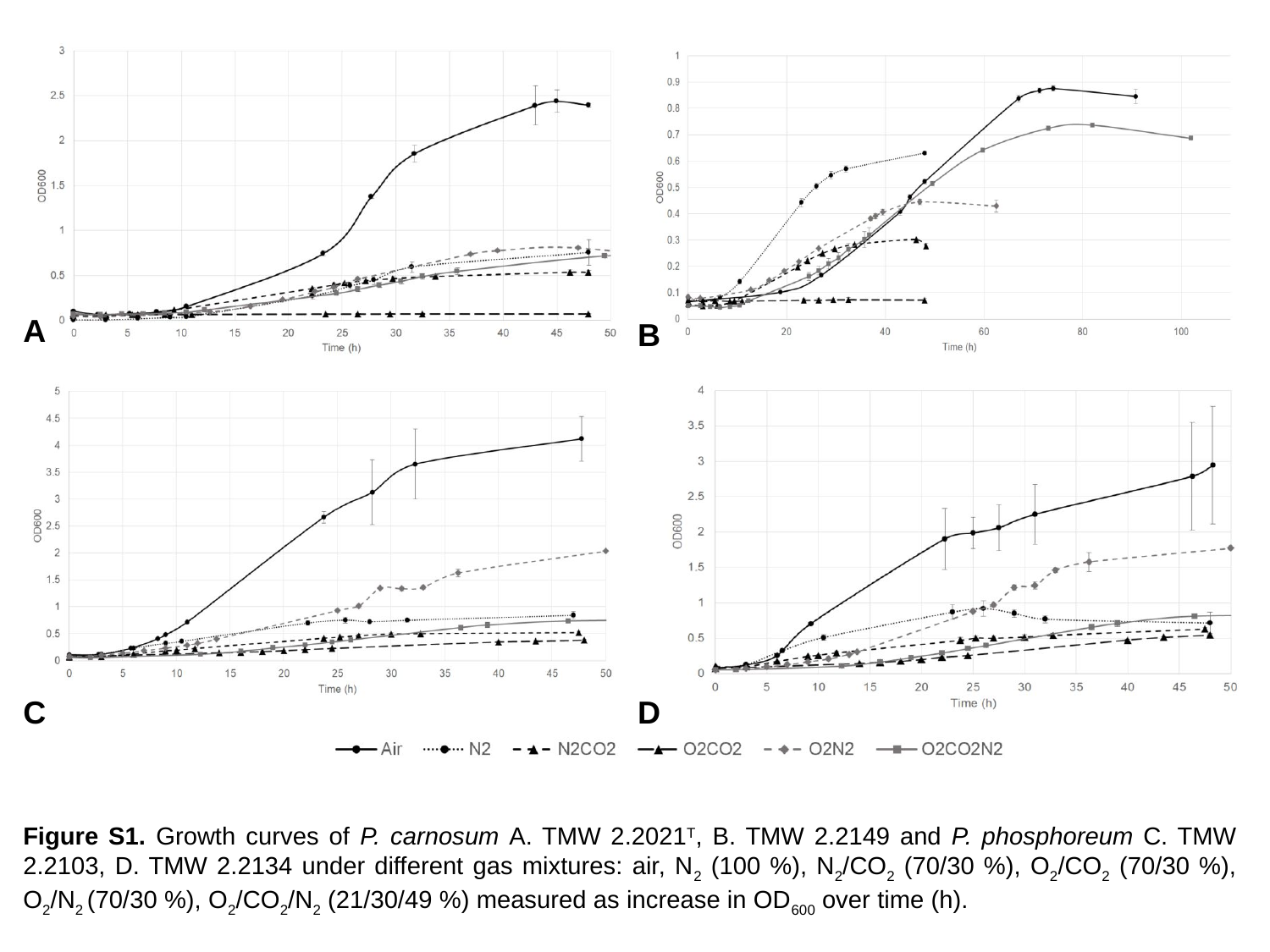

A
B
D
C
Figure S1. Growth curves of P. carnosum A. TMW 2.2021T, B. TMW 2.2149 and P. phosphoreum C. TMW 2.2103, D. TMW 2.2134 under different gas mixtures: air, N2 (100 %), N2/CO2 (70/30 %), O2/CO2 (70/30 %), O2/N2 (70/30 %), O2/CO2/N2 (21/30/49 %) measured as increase in OD600 over time (h).

## Slide 2
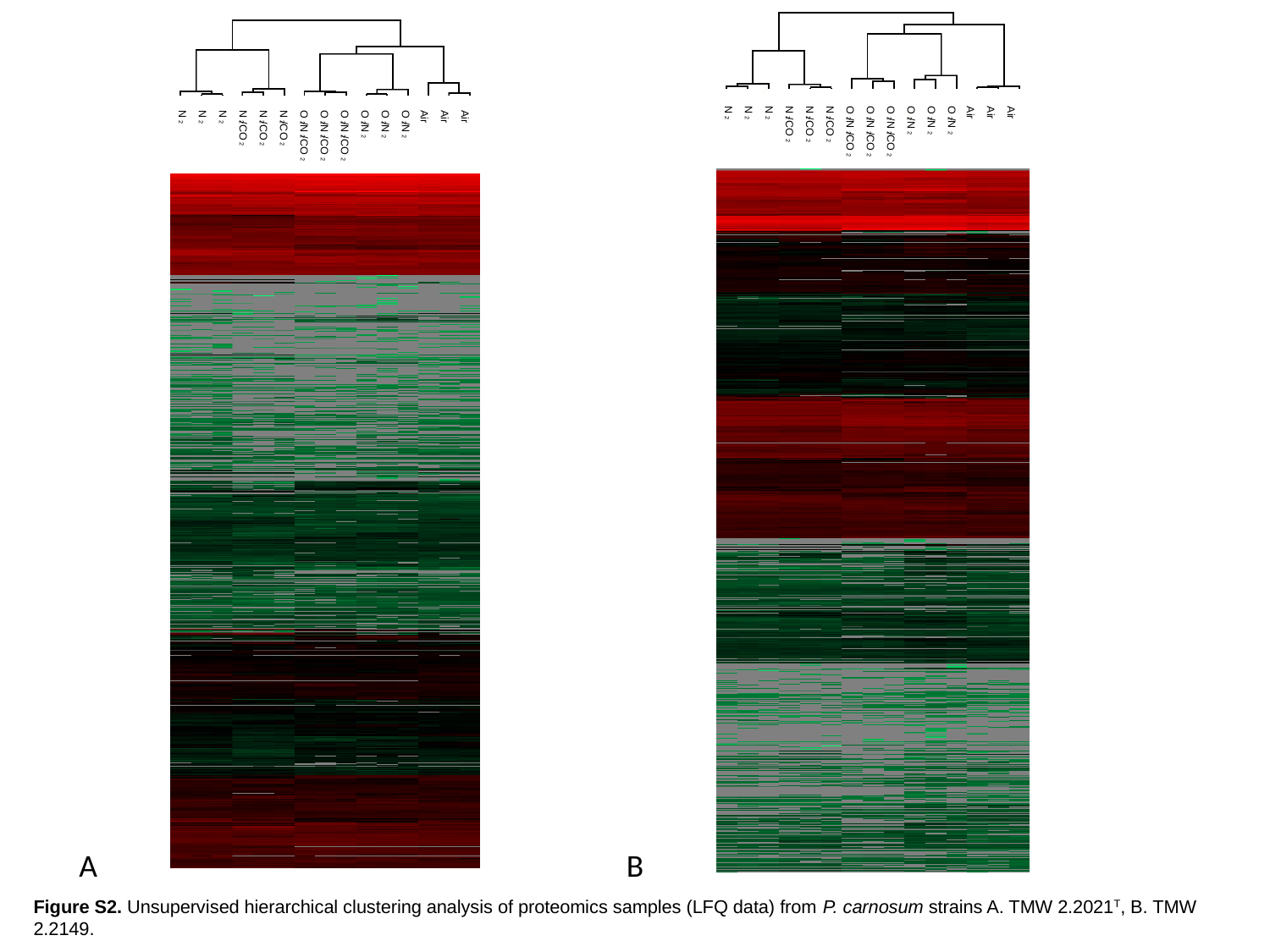

| N2 | N2 | N2 | N2/CO2 | N2/CO2 | N2/CO2 | O2/N2/CO2 | O2/N2/CO2 | O2/N2/CO2 | O2/N2 | O2/N2 | O2/N2 | Air | Air | Air |
| --- | --- | --- | --- | --- | --- | --- | --- | --- | --- | --- | --- | --- | --- | --- |
| N2 | N2 | N2 | N2/CO2 | N2/CO2 | N2/CO2 | O2/N2/CO2 | O2/N2/CO2 | O2/N2/CO2 | O2/N2 | O2/N2 | O2/N2 | Air | Air | Air |
| --- | --- | --- | --- | --- | --- | --- | --- | --- | --- | --- | --- | --- | --- | --- |
A
B
Figure S2. Unsupervised hierarchical clustering analysis of proteomics samples (LFQ data) from P. carnosum strains A. TMW 2.2021T, B. TMW 2.2149.

## Slide 3
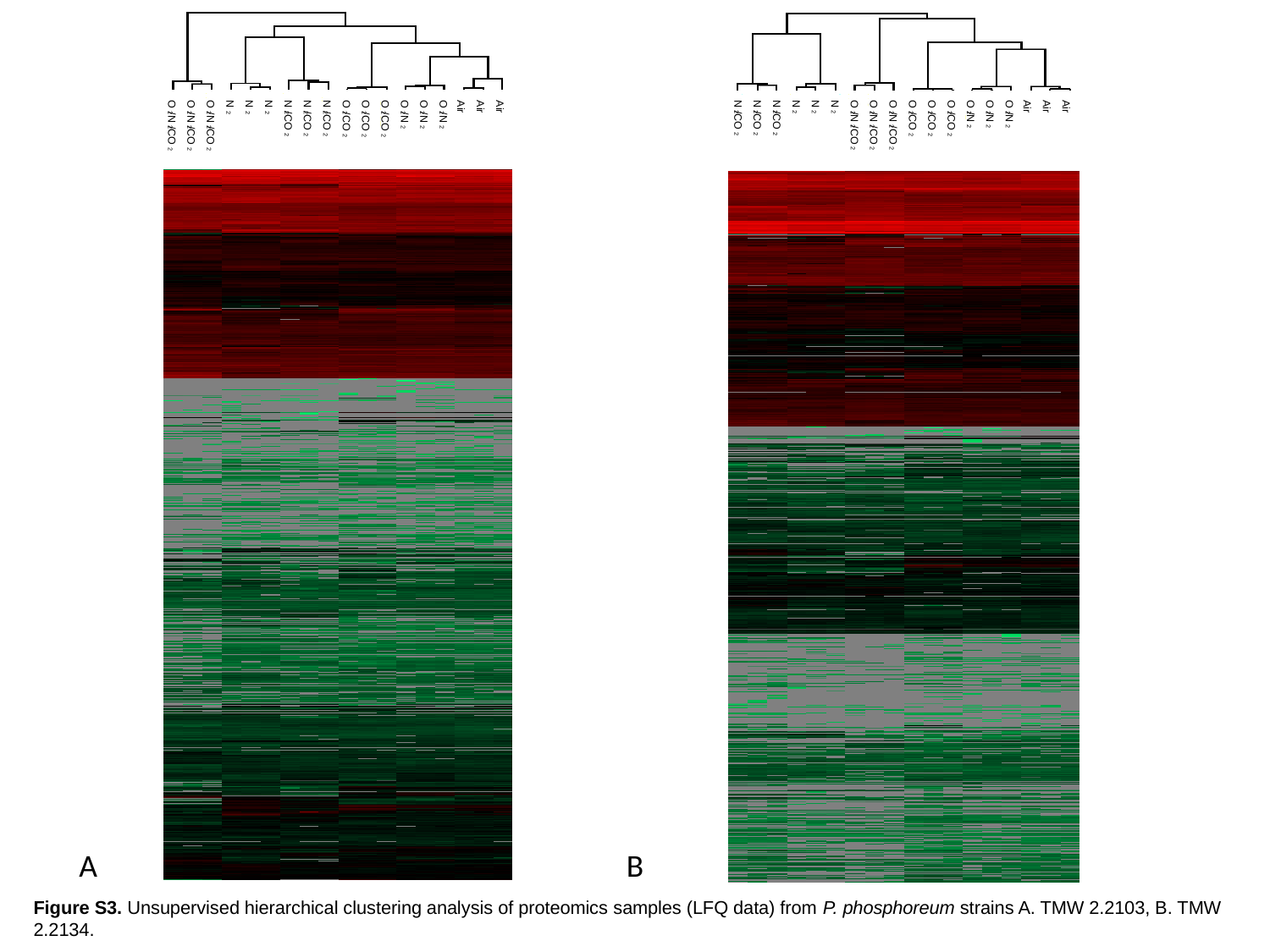

| N2/CO2 | N2/CO2 | N2/CO2 | N2 | N2 | N2 | O2/N2/CO2 | O2/N2/CO2 | O2/N2/CO2 | O2/CO2 | O2/CO2 | O2/CO2 | O2/N2 | O2/N2 | O2/N2 | Air | Air | Air |
| --- | --- | --- | --- | --- | --- | --- | --- | --- | --- | --- | --- | --- | --- | --- | --- | --- | --- |
| O2/N2/CO2 | O2/N2/CO2 | O2/N2/CO2 | N2 | N2 | N2 | N2/CO2 | N2/CO2 | N2/CO2 | O2/CO2 | O2/CO2 | O2/CO2 | O2/N2 | O2/N2 | O2/N2 | Air | Air | Air |
| --- | --- | --- | --- | --- | --- | --- | --- | --- | --- | --- | --- | --- | --- | --- | --- | --- | --- |
A
B
Figure S3. Unsupervised hierarchical clustering analysis of proteomics samples (LFQ data) from P. phosphoreum strains A. TMW 2.2103, B. TMW 2.2134.
